# Structure-activity relationship studies of four novel 4-aminopyridine K^+^ channel blockers

**DOI:** 10.1038/s41598-019-56245-w

**Published:** 2020-01-09

**Authors:** Sofia Rodríguez-Rangel, Alyssa D. Bravin, Karla M. Ramos-Torres, Pedro Brugarolas, Jorge E. Sánchez-Rodríguez

**Affiliations:** 1 Departamento de Física, Universidad de Guadalajara, Guadalajara, Jalisco 44430 Mexico; 2 Gordon Center for Medical Imaging, Department of Radiology, Massachusetts General Hospital and Harvard Medical School, Boston, MA 02114 USA

**Keywords:** Potassium channels, Ion channels in the nervous system

## Abstract

4-Aminopyridine (4AP) is a specific blocker of voltage-gated potassium channels (K_V_1 family) clinically approved for the symptomatic treatment of patients with multiple sclerosis (MS). It has recently been shown that [^18^F]3F4AP, a radiofluorinated analog of 4AP, also binds to K_V_1 channels and can be used as a PET tracer for the detection of demyelinated lesions in rodent models of MS. Here, we investigate four novel 4AP derivatives containing methyl (-CH_3_), methoxy (-OCH_3_) as well as trifluoromethyl (-CF_3_) in the 2 and 3 position as potential candidates for PET imaging and/or therapy. We characterized the physicochemical properties of these compounds (basicity and lipophilicity) and analyzed their ability to block Shaker K^+^ channel under different voltage and pH conditions. Our results demonstrate that three of the four derivatives are able to block voltage-gated potassium channels. Specifically, 3-methyl-4-aminopyridine (3Me4AP) was found to be approximately 7-fold more potent than 4AP and 3F4AP; 3-methoxy- and 3-trifluoromethyl-4-aminopyridine (3MeO4AP and 3CF_3_4AP) were found to be about 3- to 4-fold less potent than 4AP; and 2-trifluoromethyl-4-AP (2CF_3_4AP) was found to be about 60-fold less active. These results suggest that these novel derivatives are potential candidates for therapy and imaging.

## Introduction

In normally myelinated neurons, voltage-gated potassium (K^+^) channels K_v_1.1 and K_v_1.2 are clustered near the nodes of Ranvier beneath the myelin sheath^1,2^. Upon demyelination, these channels become exposed, migrate through the demyelinated segment and concomitantly increase in expression several fold^3–9^. This aberrant redistribution of K^+^ channels impairs conduction of action potentials, which leads to neurological deficits^3,10–14^. 4-aminopyridine (4AP) is a selective blocker of K_v_ channels^15–21^ used clinically to improve neurological conduction in people with multiple sclerosis (MS)^22–26^ and other demyelinating diseases^27,28^. Mechanistically, 4AP blocks the exposed K^+^ channels and therefore enhances conduction^16,17,19,20,29–32^. Recently, it has been shown that the fluorinated derivative 3-fluoro-4-aminopyridine (3F4AP) also binds to these channels^33^ and, once labeled with ^18^F, can serve to detect areas of demyelination using positron emission tomography (PET)^33–37^. Given the potential of these molecules as therapeutic and imaging agents, we set out to investigate four new 4AP derivatives and their structure-activity relationships.

**Figure 1:**
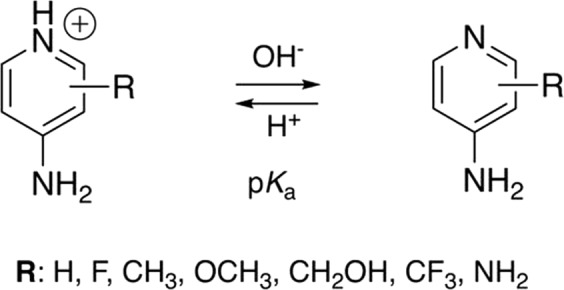
Acid-base equilibrium of 4-aminopyridine derivatives.

Prior work on the structure-activity relationship studies of 4AP derivatives has shown that small modifications on the 3 position are permitted^33,38–40^ and that the *in vivo* potency is highly correlated with the p*K*_a_^29,31^. 4AP and derivatives are basic compounds that exist in the protonated or neutral form depending on the pH of the medium (Fig. 1). The protonated form mimics a large K^+^ ion and is required to block the channel^41^, while the neutral form is required for the drug to get across the blood-brain barrier (BBB)^42^. In addition, the pharmacokinetic properties are largely dependent on the lipophilicity and p*K*_a_ of these molecules. For example, 4AP has a p*K*_a_ of 9.6, resulting in high potency but slow penetration into the CNS, which explains why a slow release formulation is required for therapy^22,23,43^. 3F4AP, on the other hand, has a p*K*_a_ of 7.6 which results in a faster CNS penetration, which is favorable for PET imaging^33^. Additional aminopyridine derivative examples include 3, 4-diaminopyridine (3, 4-DAP), a potent K_v_ channel blocker^40^ with low BBB permeability^42^, used clinically for Lambert-Eaton syndrome^44^, a disorder of peripheral nervous system, and 4-aminopyridine-3-methanol (4AP3MeOH) which has been shown to enhance conduction in laboratory models of spinal cord injury and MS^39,45^ but that has minimal permeability to the BBB and requires intrathecal administration.

**Figure 2:**
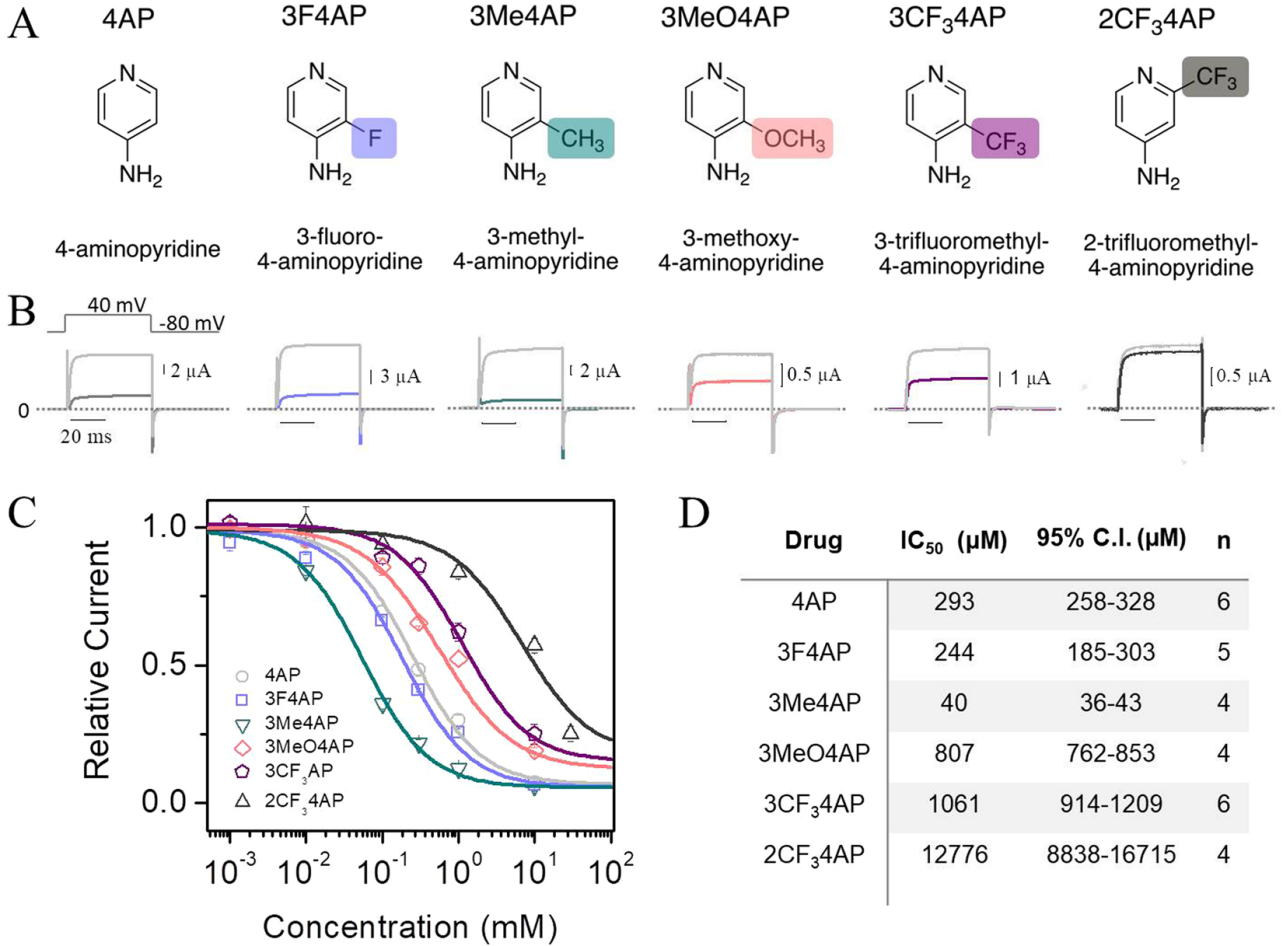
Inhibition of K^+^ currents by 4AP analogs. (**A**), Chemical structures of studied 4AP analogs. From left to right: 4-aminopyridine (4AP), 3-fluoro-4-aminopyridine (3F4AP), 3-methyl-4-aminopyridine (3Me4AP), 3-methoxy-4-aminopyridine (3MeO4AP), 3-trifluoromethyl-4-aminopyridine (3CF34AP), 2-trifluoromethyl-4-aminopyridine (2CF34AP). (**B**), Representative recordings of K^+^ current acquired from four to six different oocytes expressing the Shaker K_v_ ion channel before (gray) and after (colored line) addition of 1 mM of each 4AP analog. Currents were elicited by 50 ms depolarization steps from −100 to 50 mV in increments of 10 mV. For clarity, only the inhibition of K^+^ current by cumulative concentration of 4AP and derivatives recorded at 40 mV is shown. The dashed line indicates current at a value of zero and the 20 ms horizontal bar represents the time scale for all recordings. (**C**), Relative current as a function of the concentration of 4AP analogs obtained at 40 mV and pH 7.4. (**D**), IC_50_ of each 4AP analog and 95% confidence interval obtained by fitting the data with the Hill equation (Eq. 1). n represents the number of times each drug was tested in separate oocytes.

Given the correlations between the p*K*_a_, lipophilicity and molecular size with *in vivo* activity, we hypothesized that the derivatives 3-methyl-4-aminopyridine (3Me4AP), 3-methoxy-4-aminopyridine (3MeO4AP), 3-trifluoromethyl-4-aminopyridine (3CF_3_4AP) and 2-trifluoromethyl-4-aminopyridine (2CF_3_4AP) would be permeable to the CNS and be suitable candidates for therapy and/or imaging (Fig. 2A). These molecules are particularly interesting as potential PET radioligands since they are amenable to labeling with ^11^C, which provides some advantages over ^18^F-labeled radioligands. While ^11^C has a significantly shorter half-life compared to ^18^F (20 *vs*. 110 min) limiting its use to sites with a cyclotron, ^11^C-labeled tracers tend to be easier to radiolabel and their short half-life allows for multiple scans on the same subject and day; an important advantage during tracer development and validation. In fact, methods to produce radiolabeled [^11^C]3MeO4AP, [^11^C]2CF_3_4AP and [^11^C]3CF_3_4AP^46,47^ have recently been communicated but evidence that these compounds are able to bind to K_V_ channels is lacking.

## Results

### Basicity and lipophilicity of 4AP analogs

The chemical structures and the abbreviations of the 4AP analogs studied are shown in Fig. 2A. Table 1 shows the p*K*_a_ values of these compounds in order of decreasing basicity. As shown on the table: 4AP, 3Me4AP, and 3MeO4AP are more basic with p*K*_a_ values higher than 9, while 3F4AP, 3CF_3_4AP and 2CF_3_4AP are less basic with p*K*_a_ values lower than 8. This indicates that the former are mostly protonated at physiological pH, while the latter exist both in the protonated and neutral forms at physiological pH.

**Table 1:** p*K*_a_ and logD (at pH = 7.4) values of the 4AP analogs.

Drug	p*K*_a_	logD
3Me4AP	9.82 ± 0.06	−1.232 ± 0.008
4AP	9.58 ± 0.07	−1.478 ± 0.014
3MeO4AP	9.18 ± 0.02	−0.76 ± 0.03
3F4AP	7.65 ± 0.15	0.414 ± 0.002
2CF_3_4AP	7.0 ± 0.4	0.906 ± 0.006
3CF_3_4AP	7.17 ± 0.04	1.484 ± 0.009

In terms of lipophilicity 4AP, 3Me4AP, and 3MeO4AP were found to have octanol/water partition coefficient values at pH 7.4 of −1.48, −1.23 and −0.76 (Table 1). This indicates that these compounds preferably partition in the aqueous layer and may have lower penetration of the BBB by passive diffusion. In contrast, 3F4AP, 2CF_3_4AP and 3CF_3_4AP show partition coefficient values of 0.41, 1.48 and 0.91 (Table 1) indicating that these compounds preferably partition in the octanol layer and may have a faster permeation of the BBB. In fact, 4AP is known to have a slow penetration of the BBB while 3F4AP has a fast BBB penetration.

Both p*K*_a_ and logD trends can be rationalized by the electron-donating or electron-withdrawing nature of the substituent. As the electron-withdrawing strength of the group increases, the dipole of the pyridine nitrogen decreases resulting in a more acidic proton. When comparing the 2CF_3_4AP with 3CF_3_4AP, substitution in the 2 position results in more polar molecule. In general, the lower basicity (lower p*K*_a_) the greater the lipophilicity (higher logD) as the lower basicity results in a greater fraction of the neutral form which preferably partitions in the octanol layer. In the case of 3Me4AP, this compound is more basic than 4AP (higher p*K*_a_) and yet more lipophilic (lower logD), likely due to the lipophilicity of the methyl group compared to a proton.

### Blocking capacity of 4AP analogs

K^+^ currents were measured in *Xenopus* oocytes expressing the commonly studied voltage-gated K^+^ channel Shaker from *D. melanogaster*. In order to determine the relative blocking capacity, each drug was applied to the same oocyte at increasing concentrations ranging from 1 to 10,000 μM. Then, the relative current was determined as the ratio between the maximal amplitude of the K^+^ current in the absence and in the presence of each drug. Figure 2B shows five representative K^+^ current traces from Shaker elicited at 40 mV, before and after addition of 1,000 μM of each drug. Figure 2C shows the relative current as a function of the concentration of each 4AP analog tested. The Hill equation (Eq. 1) was fitted to the dose-response curves and used to calculate the IC_50_ for each drug at pH 7.4. Hill parameters are summarized in Fig. 2D. Our results indicate that the relative potency of blocking, from highest to lowest, of these 4AP analogs is: 3Me4AP, 3F4AP, 4AP, 3MeO4AP, 3CF_3_4AP and 2CF_3_4AP. Specifically, our results show that at pH 7.4 and voltage 40 mV 3Me4AP is approximately 7-fold more potent than 4AP, 3F4AP is comparable to 4AP, and 3MeO4AP and 3CF_3_4AP are approximately 3- and 4- fold less potent than 4AP, respectively, and 2CF_3_4AP was found to have very little capacity of blocking the channel (~50% at 10 mM). These results are in agreement with the prior observation that small modifications in the 3 position of 4AP are permitted, whereas large modifications significantly diminish the potency of blockage^33^.

### Dependence of the blocking on pH

Since the canonical mechanism describes that only positively-charged molecules can block the channel^41^ and protonation of the drug is dependent on the pH, we studied the blocking of the channel (IC_50_) at pH 6.8, 7.4 and 9.1. To avoid a gradient in pH, both internal and external solutions were replaced.

**Figure 3:**
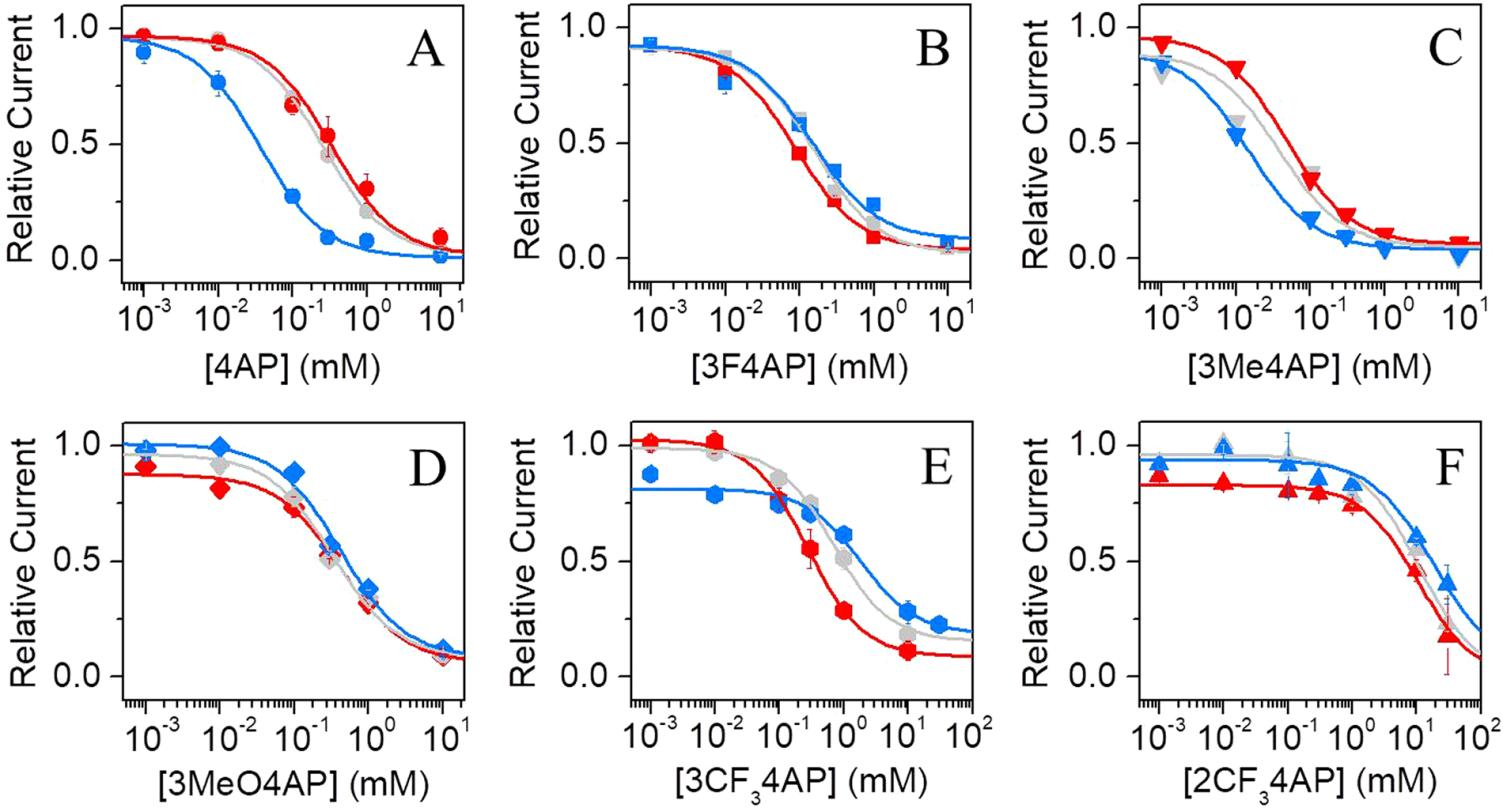
IC_50_ at 0 mV and pH dependence. Relative current *vs*. concentration at 0 mV at pH of 6.8 (red), 7.4 (gray) and 9.1 (blue) of: (**A**), 4AP, (**B**), 3F4AP, (**C**), 3Me4AP, (**D**), 3MeO4AP and, (**E**), 3CF_3_4AP, (**F**), 2CF_3_4AP. Continuous lines in panels **A** to **E** represent the fits with the Hill equation (Eq. 1). Hill parameters are summarized on Table 2.

Panels **A** to **E** of Fig. 3 show the effects on the relative current as a function of concentration for each 4AP derivative at the different pH values. Interestingly, the analogs with high p*K*_a_ (4AP, 3Me4AP and 3MeO4AP) showed higher blocking ability (lower IC_50_) at higher pH, whereas the analogs with lower p*K*_a_ showed higher blocking ability at lower pH. Since 4AP and derivatives bind from the intracellular side^16^, we hypothesize that in the case of the compounds with high p*K*_a_ the limiting factor is the permeability of the drug through the oocyte membrane at low pH. In the case of the compounds with low p*K*_a_, these drugs are able to permeate through the membrane even at low pH and the limiting factor is the fraction of protonated or active form of the drug. Table 2 summarizes the IC_50_ values of each analog at different pH values calculated by fitting the Hill equation to the data.

**Table 2:** IC_50_ values of 4AP analogs at 0 mV: Hill parameters.

Drug	pH 6.8			pH 7.4			pH 9.1		
	IC_50_ (μM)	95% C.I. (μM)	n	IC_50_ (μM)	95% C.I. (μM)	n	IC_50_ (μM)	95% C.I. (μM)	n
4AP	210	176–244	4	199	190–207	6	38	33–44	4
3F4AP	82	76–88	5	160	140–179	5	185	144–226	4
3Me4AP	66	56–76	6	39	36–42	4	15	13–17	5
3MeO4AP	357	325–390	4	355	327–382	4	492	446–538	5
3CF_3_4AP	249	150–348	5	690	633–747	6	2188	1317–3057	4
2CF_3_4AP	10,448	7,909–12,985	4	11,903	5,111–18,695	4	18,215	2776–33,653	2

### Dependence of the blocking on voltage

**Figure 4:**
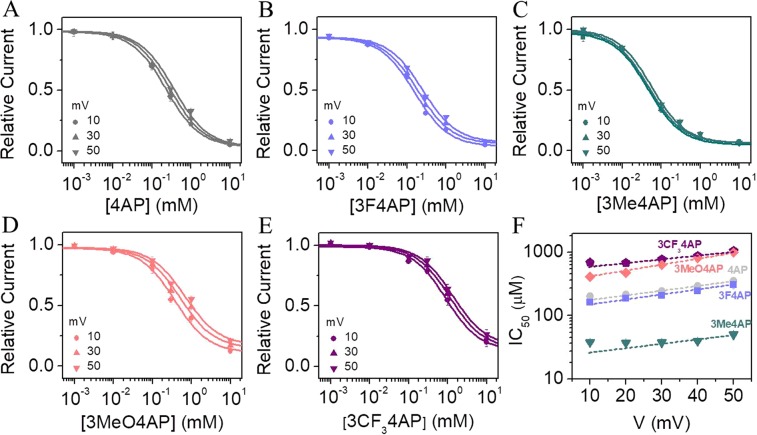
Voltage-dependence of the IC_50_ for each 4AP analog. Relative current as a function of concentration of (**A**) 4AP, (**B**) 3F4AP, (**C**) 3Me4AP, (**D**) 3MeO4AP, and (**E**) 3CF_3_4AP, obtained at different values of voltage. For clarity only the curves obtained at 10, 30 and 50 mV are shown. Solid lines represent the fits of the data with the Hill equation (Eq. 1). (**F)** IC_50_ *vs*. voltage curves of 4AP analogs determined in the range of voltage from 10 to 50 mV. IC_50_ values were obtained from the analysis of the data of the panels A to E. Dashed lines represent the fits with the Woodhull model (Eq. 2). Woodhull parameters δ and IC_50_ at V = 0 mV are shown on Table 3.

It is known that blocking of K_V_ channels by 4AP involves sequential voltage-dependent rearrangements of the protein^31^. During this process, K_V_ channels must transition to the open conformation before 4AP can bind to its site inside the channel pore. Therefore, it is expected that the blocking of the studied 4AP derivatives will be voltage-dependent as it has been shown for 4AP^48^. For this reason, we studied the IC_50_ at several voltage values. We recorded K^+^ currents from Shaker channel in the range of voltage from −100 to 50 mV (see Fig. S1) and calculated the IC_50_ of each drug and voltage as described above. Panels **A** to **E** of Fig. 4 show the measured relative current as a function of the concentration of each 4AP analog at several voltage values. For clarity, only representative curves at +10, +30 and +50 mV are shown. From Fig. 4, it can be observed that the calculated IC_50_ for each 4AP analog increased with voltage (in μM): from 200 to 350 for 4AP, from 160 to 304 for 3F4AP, from 37 to 50 for 3Me4AP, from 310 to 992 for 3MeO4AP, and from 690 to 1150 for 3CF_3_4AP. These results confirm that blocking of the channel is voltage-dependent and that it is more difficult to block the passage of K^+^ ions at higher voltages than at lower voltages.

Furthermore, this experiment allowed us to calculate the fractional distance through the membrane electrical field that each molecule travels to bind to its site during the experiment^49^. The Woodhull equation (Eq. 2) describes that the relationship between IC_50_ and voltage is dependent on δ. By fitting the IC_50_ *vs*. V curves shown in Fig. 4D to the Woodhull equation, we were able to calculate δ for each 4AP analog. The fitted parameters from this analysis are shown on Table 3. Since δ values varied between 0.41 and 0.56, we conclude that these molecules have to travel approximately across one half of the membrane electric field. The similarity of the δ values between 4AP and the other derivatives suggests that these drugs share a common binding site.

**Table 3:** IC_50_ and δ values of 4AP analogs at 0 mV: Woodhull parameters.

Drug	^*^IC_50_ (μM)	δ	n
4AP	155 ± 12	0.41 ± 0.08	6
3F4AP	122 ± 4	0.46 ± 0.10	5
3Me4AP	21 ± 1	0.43 ± 0.10	4
3MeO4AP	329 ± 17	0.56 ± 0.02	4
3CF_3_4AP	516 ± 51	0.46 ± 0.10	6

*Mean values are given ± SD.

## Discussion

Screening the ability of fluorinated analogs of 4AP to block K^+^ currents in *Xenopus* oocytes expressing Shaker K^+^ channel facilitated the identification of 3F4AP as a potential PET radioligand for demyelination. Subsequent labeling of 3F4AP with ^18^F and PET imaging studies in rodent models of MS confirmed the capacity of this compound to detect demyelinated lesions using PET^33,37^. This compound is in progress to clinical studies and has the potential to advance the imaging of demyelinating diseases.

In PET tracer development, similar to drug development, it is useful to explore multiple derivatives and compare their properties. This process provides confirmation of the target and may result in radioligands with enhanced characteristics. In addition, it is convenient to have ^18^F and ^11^C versions of the same tracer, as each radiolabeling method provides certain advantages that the other lacks. For example, while ^18^F-labeled tracers can be used in sites remote from a cyclotron because of its longer half-life, ^11^C- labeled tracers enable multiple scans (be it more than one ^11^C-scan or a combination of ^11^C/^18^F-scans) on the same subject and the same day.

Here, we studied the efficiency of blocking K^+^ currents of four novel 4AP analogs under steady-state, namely: 3-methyl-4-aminopyridine (3Me4AP), 3-methoxy-4-aminopyridine (3MeO4AP), 3-(trifluoromethyl)-4-aminopyridine (3CF_3_4AP), 2-(trifluoromethyl)-4-aminopyridine (2CF_3_4AP) (see chemical structures in Fig. 1A). We selected these compounds as are they are predicted to be permeable to the BBB and are amenable to labeling with ^11^C. During our studies, we found that all of these compounds are able to block Shaker (homolog of mammalian K_v_1.2) channel albeit with different potencies (IC_50_s ranging from 160 to 12,000 uM). Specifically, 3Me4AP was found to be ~7 times more potent than 4AP and 3F4AP, 3MeO4AP and 3CF_3_4AP were about 3–4 times less potent and 2CF_3_4AP was around 60 times less potent. This study is timely given that methods to label 3MeO4AP and 2- and 3-CF_3_4AP have recently been reported but the capacity of these compounds to bind to the K_v_ channels is unknown. Thus, this study may help decide which compounds to take for further imaging development.

The values obtained in this study represent the potency of the tested drugs towards Shaker channel expressed in Xenopus oocytes, which as discussed by A. L. Goldin is typically lower than the potency measured in mammalian cells or even native tissues^50^. These differences in potency typically arise from the large number of intussusceptions around the oocyte, the presence of the vitelline membrane, the follicles surrounding the membrane, and the intracellular yolk structures, which may act as a sink for the drug. Nevertheless, Goldin also states that the relative efficacies of drugs against channels expressed in Xenopus oocytes are generally representative of those in native tissues. Thus, this study provides strong evidence that the *in vivo* affinity of the inhibitors will be as follows: 3MeO4AP > 3F4AP > 4AP > 3MeO4AP > 3CF_3_4AP ≫ 2CF_3_4AP.

Furthermore, the physicochemical characterization of these compounds in terms of basicity and lipophilicity provided here are also informative for future prioritization in drug or tracer development as many critical properties for successful pharmaceuticals and radiopharmaceuticals are related to these properties including brain penetration, clearance rate and metabolic stability.

Finally, we studied the voltage- and pH-dependence of the compounds blocking ability, as these results can provide valuable mechanistic information. From the pH-dependence, we observed that the compounds with lower p*K*_a_ (*i.e*., 3F4AP, 3CF_3_4AP and 2CF_3_4AP) are less active at basic pH, presumably because not enough of the protonated/active form is present. At the same time, the compounds with high p*K*_a_ (*i.e*., 4AP, 3Me4AP and 3MeO4AP) are more active at basic pH as it facilitates membrane permeability. Regarding the voltage-dependence, we found that blocking is more effective at lower voltages than at higher voltages, as the voltage provides a greater driving force to the K^+^ ions than to the drugs. From the voltage-dependence analyses, we were able to estimate using the Woodhull equation that these drugs travel about 50% of the membrane electrical field in order to bind. This is consistent with prior studies about 4AP binding site^16,29^ and strongly suggests that these drugs share a common binding site.

In summary, we have characterized four novel derivatives of 4AP as potential candidates for therapy and imaging. The physicochemical and pharmacological properties described here will be useful for selecting the compounds with most potential and to explain the differences in terms of drug efficacy and tracer sensitivity.

## Methods

### p*K*_*a*_ determination

The p*K*_a_ of each compound was measured by acid titration. Briefly, 5 mg of each compound were dissolved in 5 mL of water and titrated with 0.01 M HCl solution (0.01 M NaOH solution for 2CF_3_4AP). pH was monitored with a pH meter and plotted as a function of volume of acid added to make a titration curve. p*K*_*a*_ was then found using Gran plot analysis for each replicate. This procedure was repeated 4 times for each compound.

### Partition coefficient determination

The octanol-water partition coefficient (logD) at pH 7.4 was determined using a modified version of the shake flask method. Briefly, PBS (900 µL), 1-octanol (900 µL) and a 10 mg/mL aqueous solution of each compound (2 µL) were added to a 2 mL HPLC vial. The compounds were partitioned between the layers via vortexing and centrifuged at 1,000 g for 1 min to allow for phase separation. A portion (10 µL) was taken from each layer (autoinjector was set up to draw volume at two different heights) and analyzed by HPLC. The relative concentration in each phase was determined by integrating the area under each peak and comparing the ratio of the areas from the octanol and aqueous layers. A calibration curve was performed to ensure that the concentrations detected were within the linear range of the detector. This procedure was repeated 4 times for each compound.

### Synthesis of Shaker K^+^ channel RNA

A sample of cDNA clone encoding for Shaker voltage-gated K^+^ channel from *D. melanogaster* with inactivation removed^51^ was generously provided by the laboratory of Prof. Francisco Bezanilla at The University of Chicago. The DNA was amplified, linearized with *Not I* enzyme (New England Biolabs, Inc., Ipswich, MA, USA) and transcribed *in vitro* using the T7 promoter mMESSAGE cRNA kit (Ambion, Austin, Tex., USA).

### Expression of Shaker K^+^ channels in Xenopus oocytes

Shaker channel was heterologously expressed in *Xenopus laevis* oocytes. Only mature *Xenopus laevis* frogs (Aquanimals SA de CV, Queretaro, Mexico) were used as oocytes suppliers. A volume of 1−3 mL from the ovary lobes was extracted via survival surgery under anesthesia. All methods involving live animals were performed in accordance with relevant guidelines and regulations and with the approval of the Comité Institucional del Cuidado y Uso de Animales en el Laboratorio (CICUAL-CUCEI-UDG). Subsequently, oocytes were isolated with the treatment of collagenase type II (Worthington Biochemical Corp., NJ, USA) under mechanical agitation. After the isolation with collagenase, each oocyte was injected with 15–25 ng of RNA encoding for the Shaker ion channel and incubated for 8–12 h at 17 °C in a Standard Oocytes Saline (SOS) solution containing (in mM): 100 NaCl, 1 MgCl_2_, 10 HEPES, 2 KCl and 1.8 CaCl_2_ with 50 μg/mL gentamycin at pH 7.5.

### Recording of K^+^ currents using cut-open voltage clamp

All chemical compounds for this study were acquired from Sigma-Aldrich (Sigma-Aldrich Co., St. Louis, MO, USA) and Chem-Impex International (Chem-Impex International, Inc. Wood Dael, IL, USA) unless otherwise indicated. Electrophysiology measurements were conducted using the methodology of cut-open oocyte voltage clamp (COVC)^52^. For COVC procedures, the internal recording solution contained (in mM): 120 KOH, 2 EGTA, and 20 HEPES. The external recording solution was composed (in mM) by: 12 KOH, 2 Ca(OH)_2_, 105 NMDG (N-methyl-*D*-glucamine)-methylsufonate (MES), and 20 mM HEPES. For measurements carried out at pH of 6.8 and 7.4, the pH of both solutions was adjusted with MES. For measurements carried out at pH = 9.1, HEPES was replaced by 2-(cyclohexylamino)ethanesulfonic acid (CHES).

To quantify the effects upon K^+^ currents of 4AP and 4AP analogs, oocytes that successfully expressed the Shaker channel were voltage-clamped in a COVC station. K^+^ currents were recorded in the same oocyte, first in absence (*I*_*K*_) and then in presence of each drug (*I*_*I*_). *I*_*K*_ was elicited by depolarizing the oocyte membrane with a voltage protocol that consisted in steps of 50 ms from −100 to 50 mV in increments of 10 mV. Then, *I*_*I*_ was achieved by replacing the external solution (top and guard chambers) with a solution containing increasing concentrations of each drug (4AP), 3-fluoro-4-aminopyridine (3F4AP), 3-methyl-4-aminopyridine (3Me4AP), 3-methoxy-4-aminopyridine (3MeO4AP), 2-trifluoromethyl-4-aminopyridine (2CF_3_4AP) and 3-trifluoromethyl-4-aminopyridine (3CF_3_4AP). Because 4AP and 4AP analogs block the K_V_ channels in its open conformation^31^, the oocytes were pulsed 5 to 10 times at 10 mV (1 min pulse) until a stable *I*_*I*_ was achieved. The integrity and stability of each oocyte were continuously monitored throughout the experiment.

Ionic currents were amplified and digitized with the Oocyte Clamp Amplifier CA-1A (Dagan Corporation, Minneapolis, MN, USA) and the USB-1604-HS-2AO Multifunction Card (Measurement Computing, Norton, MA, USA), respectively, and controlled with the GpatchMC64 program (Department of Anesthesiology, UCLA, Los Angeles, CA, USA) via a PC. Data were sampled at 100 kHz and filtered at 10 kHz. All the experiments were performed at room temperature (21–23 °C).

### Electrophysiology data analysis

Ion currents recordings were analyzed with Analysis (Department of Anesthesiology, UCLA, Los Angeles, CA, USA) and OriginPro 8 (OriginLab Corporation, Northampton, MA, USA.) programs. The half-maximal inhibitory concentration of 4AP and 4AP analogs (*IC*_50_) was determined by fitting the relative current (*I*_*Rel*_ = *I*_*I*_*/I*_*K*_) as a function of the cumulative concentration of each drug ([X]) with the Hill equation:

1 $${I}_{Rel}={I}_{max}+\frac{{I}_{max}-{I}_{min}}{1+{10}^{(\log I{C}_{50}-\log [X]h)}}$$

where *I*_*max*_ and *I*_*min*_ are the maximal and minimal value of *I*_*Rel*_, respectively, and *h* is the Hill coefficient, which was typically 1.0 ± 0.1 under our experimental conditions.

The voltage-dependence of blocking by 4AP and 4AP analogs was analyzed in terms of the *IC*_50_ as a function of V (*IC*_50_*(V)*). *IC*_50_*(V)* curves were fitted with a one-step model of inhibition^49,53^, which allowed to determine the fractional distance through the membrane electrical field (δ) that each 4AP analog has to cross to reach its binding site:

2 $$\log \,I{C}_{50}(V)=\,\log \,I{C}_{50(V=0)}+\frac{1}{2.303}\frac{z\delta FV}{RT}$$

where *IC*_50*(V*_ _=_ _*0)*_ is the value of *IC*_*50*_ at V = 0 mV, *F* is the Faraday constant, *R* is the gas constant, *T* is the ambient temperature, and z is the apparent charge. Mean values of data ± standard deviation (s.d.) are given or plotted and the number of experiments is denoted by n. The 95% of confidence interval (IC_95_) is denoted as [Upper limit-Lower limit]; where Upper limit = ${10}^{({\rm{\log }}I{C}_{50}+s.d)}$ and Lower limit = ${10}^{({\rm{\log }}I{C}_{50}-s.d)}$.

## Supplementary information

Supplementary Information

## Data Availability

The datasets generated during and/or analyzed during the current study are available from the corresponding author upon reasonable request.
