# Supplementary material for: Structure-activity relationship studies of four novel 4-aminopyridine K+ channel blockers
Source: Sci Rep. 2020 Jan 9;10:52. doi: 10.1038/s41598-019-56245-w (PMC6952366; doi:10.1038/s41598-019-56245-w)
Supplement: Supplementary file 1 — Supplementary Information [file 41598_2019_56245_MOESM1_ESM.pdf]

Supporting Information:

Structure-activity relationship studies of four novel 4-aminopyridine K^+ channel blockers

Sofia Rodríguez-Rangel[1], Alyssa D. Bravin[2], Karla M. Ramos-Torres[2], Pedro Brugarolas[2*], Jorge E. Sánchez-Rodríguez[1*]

[1] Departamento de Física, Universidad de Guadalajara, Guadalajara, Jalisco 44430, Mexico.

[2] Gordon Center for Medical Imaging, Department of Radiology, Massachusetts General Hospital and Harvard Medical School, Boston, MA 02114.

*** Corresponding authors**

Jorge E. Sánchez-Rodríguez, PhD, Laboratorio de Biofísica Molecular, Departamento de Física, Universidad de Guadalajara, CUCEI, Blvd. Marcelino García Barragán 1421, C.P. 44430, Guadalajara, Jalisco, México; email: jorge.srodriguez@academicos.udg.mx.

Pedro Brugarolas, PhD, Gordon Center for Medical Imaging, Massachusetts General Hospital and Harvard Medical School, Department of Radiology, 55 Fruit St, Bulfinch building, Room 051 Boston, MA 02114; email: pbrugarolas@mgh.harvard.edu.

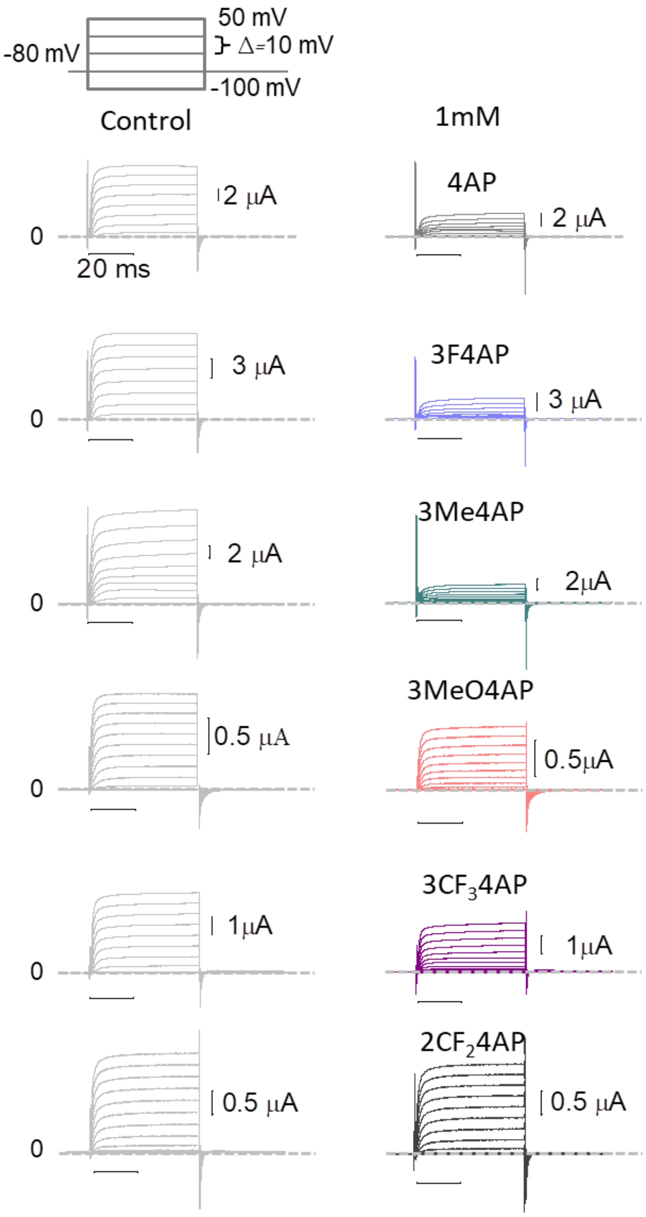

Figure S1. Blockage of K^+ currents by 4AP analogs. Representative recordings of K^+ current generated by the Shaker K_V ion channel expressed in *Xenopus* oocytes, before (gray) and after addition of 1 mM of each 4AP analog (color line): 4-aminopyridine (4AP), 3-fluoro-4-aminopyridine (3F4AP), 3-methyl-4-aminopyridine (3Me4AP), 3-methoxy-4-aminopyridine (3MeO4AP), 3-(trifluoromethyl)-4-aminopyridine ($3CF_34AP$), 2-(trifluoromethyl)-4-aminopyridine ($2CF_34AP$). Currents were evoked applying the voltage protocol shown in the upper left of the figure. The gray dash line represents the zero current level.
